# Community-engaged COVID-19 contact tracing initiative in Chicago

**DOI:** 10.1017/cts.2026.10744

**Published:** 2026-05-06

**Authors:** Sage J. Kim, Noah McWhirter, Sangeun Lee, Ajanta Patel, Jeni Hebert-Beirne, Ronald Hershow, Emily Stiehl

**Affiliations:** 1 University of Illinois Chicago School of Public Health, Chicago, IL, USA; 2 School of Nursing, University of Wisconsin–Milwaukee, Milwaukee, WI, USA; 3 Chronic Disease Prevention and Health Promotion, Chicago Department of Public Health, Chicago, IL, USA

**Keywords:** COVID-19, contact tracing, community-based organizations, infection control

## Abstract

**Introduction:**

During the early days of the COVID-19 pandemic, the Chicago Department of Public Health (CDPH) partnered with 31 community-based organizations (CBOs) to implement contact tracing to identify and manage potential COVID-19 infection cases in the city of Chicago.

**Methods:**

To evaluate the performance of the COVID-19 contact tracing, we utilized data between September 16, 2020, and December 31, 2021. Contact tracers collected data through phone interviews with potentially affected individuals. The level of initiation, timeliness, and completion of the contact tracing process were examined.

**Results:**

A total of 38,086 unique individuals were included in the analysis. Compared to White contacts, Blacks were more likely to refuse contact tracing, and Hispanics were less likely to be contacted within 7 days of exposure. Community areas with a greater number of contact tracing cases had a lower proportion of call completion.

**Conclusions:**

There was significant difficulty conducting contact tracing in the early months of COVID-19 due to the high volume of infected individuals. Contact tracing efforts were less successful among racial/ethnic minority residents despite the city’s efforts to hire and train community members as contact tracers by engaging a wide range of CBOs. Preparedness plans for future pandemic events will benefit from strategies to improve community response to surveillance programs.

## Introduction

Contact tracing is a public health surveillance tool used to identify, assess, and manage individuals who may have been exposed to infectious diseases by an infected person [1]. During the COVID-19 pandemic, the large volume of cases quickly overwhelmed existing infrastructure, which prompted local health departments to rapidly hire and train a new workforce to support contact tracing. Health departments developed unique models to hire contact tracers, train them for the job, organize the workers, and monitor the effectiveness of contact tracing efforts.

The effectiveness of contact tracing can be evaluated by whether the spread of disease is slowing down because of the contact tracing effort [2]. In terms of the implementation of contact tracing, the performance evaluation can include two key outcomes: completion and the timeliness of contact tracing [3]. However, the success of contact tracing largely depends on how people respond to contact tracing outreach, including the attitudes, perceptions, and practices around receiving calls and engaging in public health interventions [4–6]. The level of engagement in contact tracing is also associated with the level of trust in institutions, perceived personal benefit, and community engagement. Mistrust of the government and healthcare system, unmet needs, and fear of stigmatization are shown to be barriers to participating in contact tracing [7, 8]

The Chicago Department of Public Health (CDPH) launched Chicago contact tracing, known as “ChiTracing,” in September 2020. To build community trust, CDPH engaged 31 local community-based organizations (CBOs) to recruit, hire, and train community members as contact tracers. These CBOs were selected from Chicago’s priority areas, which were community areas disproportionately affected by the pandemic. Each CBO employed 15–20 contact tracers, totaling over 600 contact tracers working for ChiTracing. CDPH collaborated with the CBOs to train contact tracers. Contact tracer training covered increasing knowledge about COVID-19, infectious disease outbreaks, reducing the spread of disease, and vaccines, as well as fostering relationships and building trust. Training also included conducting contact tracing calls and creating referrals for social needs [9].

The purpose of this paper is to evaluate the early effectiveness of the ChiTracing implementation and to examine differences in ChiTracing participation and completion by race/ethnicity and residential community areas. We then discuss the challenges and limitations of our experience implementing ChiTracing. This analysis includes a large number of cases encompassing all contacts recorded in the city-run contact tracing efforts, along with individual demographic information.

## Materials and methods

ChiTracing contact tracers were responsible for calling individuals who were identified as close contacts by COVID-19 positive cases. ChiTracing reached out to individuals who: (1) were identified as a close contact who came in contact with someone who had tested positive for COVID-19 within 6 feet for more than 15 minutes, (2) resided in Chicago, and (3) had an available telephone number. Individuals were excluded if they: (1) were under the age of 18 years, (2) had COVID-19 positive test results prior to contact tracing outreach, or (3) were outside of the jurisdiction, including contacts that were referred to the CDPH Congregate Settings Team (CST). Contacts completed an initial interview by phone and were enrolled in monitoring, which lasted up to two weeks. Some contacts agreed to participate in the contact tracing and completed the initial intake form, while others refused to participate or were unreachable.

Contact tracers were provided with call scripts that included an introduction and gathering information about the contact, such as race/ethnicity, gender, sexual orientation, residence, working and living settings, symptoms, and existing health conditions. For example, the introduction script was: *“Hello, my name is [Interviewer Name] and I am an investigator calling from the Chicago Department of Public Health to discuss an important health matter.”* Further, contact tracers were given scripts for COVID-19 education: *“As part of an investigation into a confirmed case of COVID-19, we are following up with individuals who may have had contact with a case while they were possibly contagious. I am calling to check on you and discuss some Public Health recommendations with you.”* Contact tracers also described what the call was about and ensured confidentiality: “*During this call, I will ask you questions about your health and provide you with useful information on how to access some of the services you may need… All information you share with me will be confidential… and will not be shared with persons not involved in your care.”* Regarding COVID-19 tests, contact tracers asked about the location, date, type of test (mouth/nose swab, blood test, other, or unable to answer), and the result (positive, negative, indeterminate, pending, unknown).

If the result was positive, contact tracers made a referral to the case investigator for follow-up: “*Because you’ve tested positive for COVID-19, I am going to connect you with one of our case investigators, who will ask you some questions about your illness and who you may have come in contact with while infected.”* If the result was negative, the script indicated: “*You can develop COVID-19 up to 14 days after exposure to the virus, so your negative test doesn’t necessarily mean that you won’t get the disease. You should continue to self-quarantine and monitor your own health, and we will continue to check in with you until your monitoring period is over.”*

For asymptomatic contacts, quarantine instructions were provided: *“Since we don’t know yet if you will develop COVID-19, you will need to self-quarantine for 14 days from the day when you were exposed to the virus. Self-quarantine means that you will have to stay at home until [monitoring end date]… If at all possible, you should avoid contact with people at higher risk for severe illness (unless they live in the same home and had the same exposure as you) and regularly wash your hands.”* Those with symptoms received isolation instructions. Additionally, resources for mental health and other needs were provided.

All contact tracing call records from September 16, 2020, when ChiTracing began, through December 31, 2021, were obtained from the Salesforce platform. Duplicate call records were removed. Contacts could be called multiple times, and their status was updated as additional information became available. We included the most recent status. Any calls designated as pending on December 31, 2021, were excluded from the analysis due to censored call results. A total of 38,086 contact intake records were collected for this analysis. The call records included the call disposition, the number of individuals that contacts interacted with, the need for social services, demographic characteristics, and residential zip code. The project protocol was reviewed and determined to be non-human subjects research by the University of Illinois Chicago (UIC) Institutional Review Board (IRB#: 2020-1,044), since it constituted an evaluation of the program.

### Measures

Contact tracing call disposition was grouped into four mutually exclusive categories: completed, refused, incomplete, or excluded (Supplemental Table 1). Completed calls were contacts who completed the ChiTracing intake call. Contacts who refused to participate were grouped as refused. Incomplete calls included contacts who were unreachable, had the wrong phone number, or had no phone number. If a contact had been called at least three times over four days, with at least five hours between attempts, the call was designated as “administrative closure,” which was also coded as an incomplete call. Excluded calls were pending calls, contacts who resided outside the Chicago jurisdiction, or contacts who lived in congregate settings, and thus referred to the CDPH CST. We also excluded hospitalized or potentially deceased cases, which were originally categorized as incomplete, from this analysis. The timeliness of contact tracing calls was determined by whether contact was made within seven days (yes vs. no).

Contact demographic characteristics included self-reported age, gender, race/ethnicity, and residential zip code. We converted zip codes into 77 Chicago community areas. Age was grouped into four categories: 18–24 years, 25–44 years, 45–64 years, and 65 and older. The gender groups were male and female. Race/ethnicity was grouped into: White, Black, Hispanic, and Other (Asian, American Indian/Alaska Native, and Native Hawaiian/Pacific Islander).

### Analysis

We used Stata SE 18 (StataCorp, College Station, Texas, USA) for analysis. Descriptive statistics were used to explore call disposition by demographic characteristics. Chi-square and t-tests were used. Multiple logistic regressions were used to examine the call completion and timeliness in relation to demographic characteristics. The results were reported as odds ratios (OR) with 95% confidence intervals (CI). In all analyses, the level of statistical significance was considered to be *p* < 0.05. ArcGIS Pro was used to visualize rates of call completion by the 77 Chicago Community Areas.

## Results

**Figure 1.**
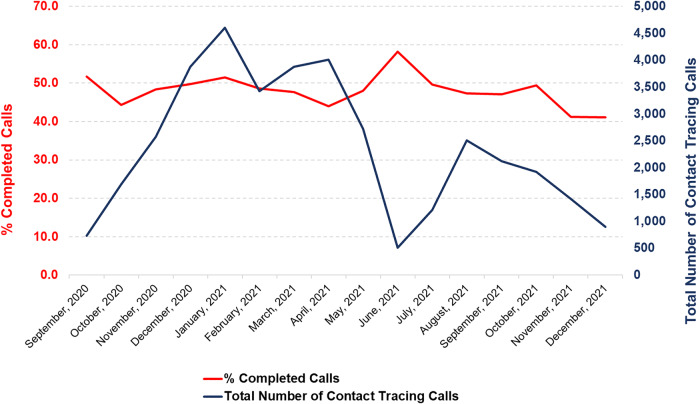
Average monthly number of contact tracing calls and % completed calls between September 2020 and December 2022. *Note:* % completed calls over time (in red) is presented on the left Y axis; the total number of contact tracing calls per month (in blue) is presented on the right Y axis.

The average monthly number of contact tracing calls and the call completion rate between September 2020 and December 2021 are shown in Figure 1. The total number of contact tracing calls gradually increased from just over 1,000 calls in September 2020 to more than 8,000 calls by April 2021. After April 2021, the number of calls dropped dramatically, peaking at slightly below 5,000 calls during the remaining months of the study.

Table 1 describes the demographic characteristics of 38,086 contacts included in the analysis. Those with missing values for race/ethnicity were excluded. Of the 38,086 call records, 21.9% were White, 25.1% were Black, 42.1% were Hispanic, and 11.0% were Other races, including Asian, American Indian/Alaska Native, and Native Hawaiian/Pacific Islander. Overall, 48.3% of the contacts were between 25 and 44 years of age, and an additional 28.4% were between 45 and 64 years old. There was a statistical difference in age distribution by race/ethnicity (*p* < 0.01), where a smaller proportion of White contacts (12.7%) and the largest proportion of Hispanic contacts (18.3%) were in the youngest age group, between 18 and 24 years. On the other hand, 6.1% of Other races and 6.4% of Hispanic contacts were 65 years and older, while 10.3% of Black contacts were 65 and older. A significantly lower proportion of Black (35.9%) contacts were male than in other race/ethnic groups (>45%).

**Table 1.** Demographic characteristics of chiTracing contacts

		Race/Ethnicity					
Variable		Total	White	Black	Hispanic	Other^a^	*p*
**Age**	*N*	26,208	5,645	6,595	11,231	2,737	
18–24	%	15.8	12.7	14.6	18.3	14.9	<0.001
25–44		48.3	51.0	46.0	47.3	52.5	
45–64		28.4	29.3	29.0	28.0	26.5	
≥65		7.5	7.1	10.3	6.4	6.1	
**Gender**	*N*	21,148	5,248	6,452	6,997	2,451	
Female	%	57.4	54.8	64.2	54.6	53.1	<0.001
Male		42.6	45.2	35.9	45.4	46.9	
**Call disposition**	*N*	38,086	8,327	9,553	16,018	4,188	
Completed	%	47.9	51.1	49.0	45.5	48.0	<0.001
Refused		6.4	6.4	7.8	5.5	7.2	
Incomplete		45.7	42.5	43.3	49.0	44.8	
**Call timeliness**	*N*	16,159	3,869	4,426	6,957	907	
≤7 days	%	44.4	46.0	45.6	42.6	46.0	<0.001
>7 days		55.6	54.0	54.4	57.5	54.0	

a Other category includes: Asian, American Indian/Alaska Native, and Native Hawaiian/Pacific Islander.

Overall, 47.9% of the contact calls were complete, 45.7% were incomplete, and 6.4% were refused calls. A larger proportion of White contacts (51.1%) completed the contact tracing process, while less than 50% of all other groups completed the process. On the other hand, a larger proportion of Black contacts (7.8%) refused to participate, compared to Hispanics (5.5%). Conversely, a larger proportion of Hispanic contacts (49.0%) was incomplete, compared to Whites (42.5%) and Black (43.3%). Regarding timeliness, 42.6% of Hispanic contacts were contacted within seven 7 days, compared to 46% of all other groups.

Table 2 summarizes the results of multinomial logistic regression with 3 categories: completed, incomplete, and refused calls. Odds ratios for refused and incomplete calls, compared to complete calls, were presented. The odds of refusing the call, compared to completed calls, were significantly higher for older age groups compared to the youngest group (18–24 years). Further, the odds of call refusals gradually increased from 1.40 in the 25–44 age group to 2.09 in the 65 and older age group. Black contacts, compared to Whites, were 42% more likely to refuse to complete the call.

**Table 2.** Multinomial logistic regression of call completeness (*N* = 13,777)

Variable	OR	(95% CI)	*p*
Refused vs completed			
**Intercept**	–	–	<0.01
**Age**			
18–24^a^	–	–	–
25–44	1.40	(1.08, 1.82)	0.01
45–64	1.45	(1.10, 1.92)	<0.01
≥65	2.09	(1.48, 2.96)	<0.01
**Gender**			
Female^a^	–	–	–
Male	1.14	(0.97, 1.33)	0.12
**Race/Ethnicity**			
White^a^	–	–	–
Black	1.42	(1.16, 1.73)	<0.01
Hispanic	1.18	(0.93, 1.42)	0.20
Other	1.06	(0.74, 1.51)	0.77
Incomplete vs completed			
**Intercept**	–	–	<0.01
**Age**			
18–24^a^	–	–	–
25–44	0.88	(0.79, 0.98)	0.02
45–64	0.88	(0.78, 0.99)	0.04
≥65	1.14	(0.97, 1.35)	0.12
**Gender**			
Female^a^	–	–	–
Male	1.21	(1.12, 1.31)	<0.01
**Race/Ethnicity**			
White^a^	–	–	–
Black	1.26	(1.14, 1.39)	<0.01
Hispanic	1.53	(1.38, 1.68)	<0.01
Other	1.11	(0.94, 1.31)	0.24

OR: Odds Ratio; CI: Confidence interval.

a Reference group for statistical comparison.

The odds of incomplete calls were higher for the 25–44 years (OR = 0.88) and the 45–64 years (OR = 0.88) age groups than the 18–24 age group. Additionally, male contacts were 21% more likely than female contacts to have incomplete calls (*p* < 0.01). Compared to White contacts, Blacks were 26% more likely (*p* < 0.01), and Hispanics were 53% more likely to have an incomplete call (*p* < 0.01).

Table 3 summarizes the logistic regression results for the timeliness of contact tracing calls, measured as the proportion of calls initiated within seven days of exposure. Less than half of the completed calls (44.6%) were made within seven days of the initial contact. A significant association was observed between age and the timeliness of initial contact. Odds of timely call completion were 0.85 for the 25–44 group, 0.86 for the 45–64 group, and 0.76 for the 65 and older group. There was no difference in timeliness between male and female contacts *p* = 0.98). Hispanic contacts were 13% more likely, compared to Whites, to complete a call within seven days of exposure (*p* = 0.04). However, the odds of timely call completion did not statistically differ between Black and White contacts (*p* = 0.76).

**Table 3.** Logistic regression of call timeliness (*N* = 7,565)

Variable	OR	(95% C I)	*p*
**Intercept**	–	–	<0.01
**Age**			
18–24^a^	–	–	–
25–44	0.85	(0.74, 0.97)	0.02
45–64	0.86	(0.74, 1.00)	0.05
≥65	0.76	(0.61, 0.94)	0.01
**Gender**			
Female^a^	–	–	–
Male	1.00	(0.19, 1.10)	0.98
**Race/Ethnicity**			
White^a^	–	–	–
Black	0.98	(0.87, 1.10)	0.76
Hispanic	1.13	(1.01, 1.27)	0.04
Other	1.04	(0.85, 1.27)	0.70

CI: Confidence interval; OR: Odds Ratio.

a Reference group for statistical comparison.

**Figure 2.**
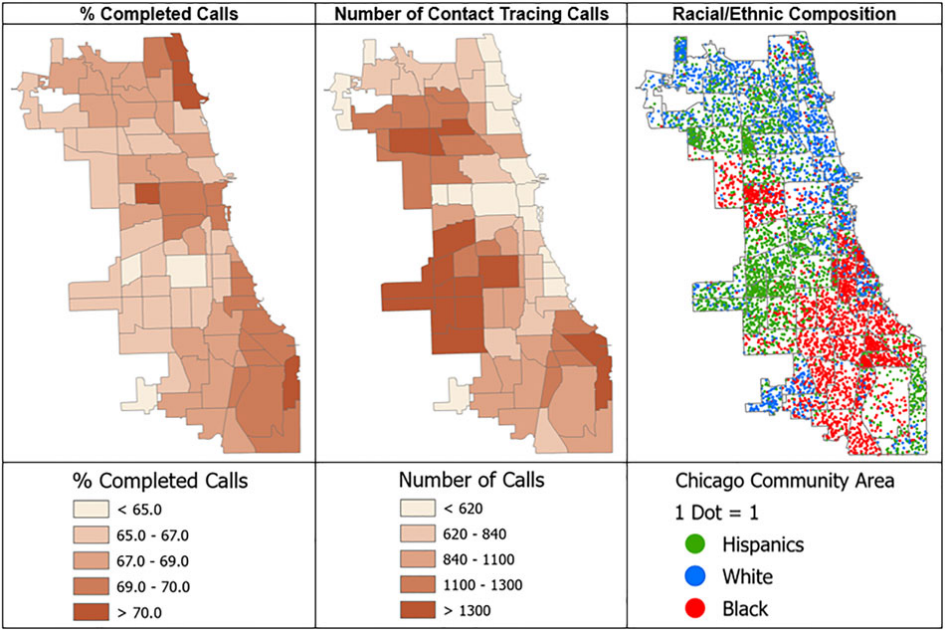
The spatial distribution of % completed calls, the number of contact tracing calls, and racial/ethnic composition by Chicago community area. *Note:* Gray borders indicate the 77 Chicago Community Areas; darker colors imply a higher % completed calls and a greater number of calls. The racial/ethnic composition map shows the proportional density of Hispanic, White, and Black residents within each community area.

Figure 2 shows the geographic distribution of call completion rates and the number of contact tracing calls made across 77 Chicago Community Areas, along with the racial/ethnic composition of the area. Communities with a greater number of contact tracing calls had a lower rate of call completion (*r* = −0.40, *p* < 0.01). These community areas were predominantly Hispanic communities (*r* = −0.34), *p* < 0.01).

## Discussion

This analysis utilized over 38,000 contact cases with demographic information, including age, race/ethnicity, and residential location, from Chicago’s city-wide contact tracing efforts during the early years of the COVID-19 pandemic. Using the unique data, we examined differences in the completion of contact tracing by age group and race/ethnicity. Furthermore, we explored the geographic distribution of contacts and completion rates in the city. The goals of this analysis were to evaluate the effectiveness of ChiTracing, which was implemented based on collaboration with CBOs across the city. We explored differences in contact tracing call completion and timeliness by demographic characteristics of contacts.

First, the successful contact tracing rate in Chicago was 47.9%, which was much lower than the initial World Health Organization (WHO) recommendation that specified 80% of new cases to be traced and quarantined within 72 hours of case confirmation for contact tracing to be considered effective [10]. Furthermore, as the incubation period shortened over time, tracing efforts needed to be more prompt [2, 11]. In our study, we examined all contacts, not confirmed cases. Still, the contact tracing rate was substantially lower than the recommended 80% standard, especially given that our cut point was based on within 7 days. Timely communication with contacts remains important for disrupting transmission [1]. A study in New Zealand suggests that the effective reproduction number was reduced when contacts were traced within 4 days [12]. As the incubation period shortened over time, tracing efforts likely needed to be more prompt [2, 11].

At the same time, the success of tracing efforts may not depend only on swift contact outreach but also on the response of community members. Given the extensive federal, state, and local messaging campaigns about COVID-19, the widespread availability of testing, and eventually, testing mandates for entering public spaces, it is possible that community members received information about what to do from sources beyond contact tracers. In particular, ChiTracing prioritized investing resources in trusted local CBOs to support the most vulnerable communities and foster collaboration [9]. And yet, we found that Black contacts were more likely to refuse calls compared to Whites, and Hispanics were more likely than Whites to have incomplete calls. Our finding is consistent with prior reports highlighting similar challenges in conducting contact tracing efforts within Black communities during the COVID-19 pandemic [13, 14]. Often, scholars discuss the pervasive distrust of government and health authorities born out of historical experiences, such as the Tuskegee trials continue to influence Black communities [15, 16], which may have resulted in a refusal to participate in contact tracing among Black Chicagoans. Similarly, the high rate of incomplete calls in the Hispanic community may be because of communication issues associated with low English proficiency, hesitancy in accessing healthcare due to citizenship status, and fear of immigration-related consequences [4, 17]. Since the initial implementation of ChiTracing, one of the CDPH’s goals has been to overcome mistrust. For this reason, non-public health individuals who were considered trusted messengers from the community were recruited and trained to conduct contact tracing, which was based on the assumption that these trusted messengers would bridge between public health and the community. However, structural and social factors affecting minority communities [18–21] may not be fully overcome with trusted messengers and messages.

Relatedly, contact tracers were hired from a wide range of CBOs that may or may not have experience working with public health. Some CBOs had strong working knowledge and infrastructure to carry out public health interventions, while others did not. Over the years, CDPH has developed strong collaborative relationships with CBOs across the city. With the existing collaborative infrastructure, CDPH’s ChiTracing aimed to fully engage a wide range of CBOs, from healthcare, youth and family services, to employment and food, to address barriers to contact tracing [9]. CBOs without public health experience had to quickly acquire the necessary basic knowledge about health, which might have been an undue burden on already resource-limited CBOs. Especially in a pandemic that demanded rapid responses, focusing on CBOs with experience implementing public health interventions would have been more effective, notwithstanding the goal of building community.

On the other hand, contact tracers introduced themselves as part of CDPH, but did not highlight their affiliation with their community and/or CBOs. If contact tracers sought out to establish local connections, the call completion rate might have been improved [4]. This is interesting because CDPH’s initial goal was to match contact tracers with contact calls based on their community. But it was not fully implemented with heavy call loads and other logistical limitations. Contact tracer’s community connection and trust building may need further investigation, which could help build trusted messengers [22].

Age and gender differences also played a major role in the success of contract tracing call completion. Older Chicagoans, especially those aged 65 and older, were more likely than younger individuals to refuse participation in contact tracing, and less likely to complete calls. This finding was unexpected, given that older individuals were shown to have higher compliance with COVID-19 recommendations due to their own perceived increased risk [23]. One reason for poor completion among older adults may be difficulty engaging with technology. The “elderly paradox” describes older adults’ lack of access to and utilization of technologies that help manage COVID-19, despite the greater needs [24–26]. Our findings suggest that efforts to support older adults in interacting with technology may help achieve better engagement with public health interventions.

Men were also less likely than women to complete contact tracing. Our finding aligns with the previously observed gender difference in healthcare utilization, where men are generally less likely than women to seek care and engage in it when it is offered [27]. This finding was particularly concerning because men were not only more likely to develop severe symptoms during the acute phase of COVID-19 infection but also die at higher rates from the disease, with nearly 2.4 times higher case-fatality compared to women [28–31]. Reasons men may avoid healthcare include a desire to avoid uncertainty, fear of discovering health issues, and a preference for self-reliance [32, 33]. Accordingly, reaching men may require different strategies, such as workplace-based interventions, prioritizing autonomy, and engaging partners and families. [28] Additionally, flexible hours and platforms, including telehealth and digital tools, would improve access to care among men [34].

### Limitations

This analysis has several limitations. First, our data covers 16 months of the pandemic, from September 2020 to December 2021, which was a period of the highest number of daily cases in Chicago, reaching over 3,000 cases per day in November 2020. While our analysis contributes valuable insights to the existing literature on pandemic responses, it also offers a snapshot of contact tracing efforts in areas with a very high number of infections. Additionally, due to challenges of launching a new program with a new data infrastructure, contact tracing call records were often incomplete and generally not in the cleanest form. We spent the first few months of 2020 hiring, onboarding, and training contact tracers, and simultaneously, testing and implementing the contact tracing case management platform. This involved a steep learning curve that included troubleshooting emergent issues with the platform and disseminating best practices through peer-learning groups of contact tracers. Regardless, our study provides a large and one of the most comprehensive data for understanding the initial pandemic response in the third-largest city in the U.S.

Second, ChiTracing data collection relied on self-reports of demographic characteristics, symptoms, and medical conditions, and we did not have a mechanism to verify the accuracy of the self-reported information by linking the ChiTracing surveillance database with city-wide testing results and medical records. Relatedly, many cases had missing demographic information, which were excluded from our analysis. This missing value problem might have affected the results of this study. Particularly, if these cases with missing information were those who did not want to be identified due to, for example, their immigration status, the distribution of call completion status as the outcome could have been affected. For example, our findings showed that Hispanic contacts were more likely to be incomplete cases, and this result could have been even more pronounced if we had included cases with missing information on race and ethnicity.

Third, ChiTracing was solely based on phone calls, which may have introduced selection biases in determining the call disposition for those who did not have working phones, which were categorized as incomplete calls. Email or other communication strategies could have been employed [35]. However, it is difficult to know how many individuals without phones actually have access to other communication applications. Nevertheless, the phone-based contact tracing may have inflated the likelihood of incomplete calls for those without phones, who are often disadvantaged and/or older individuals.

Finally, contact tracers were required to regularly adapt their messages to changing COVID-19 response policies, including guidelines on mask use, social distancing, and the emergence of new variants. The policies were often externally imposed, but solutions for adapting to the policies were co-developed by all partners to ensure the best available solutions for the local environment. This mismatch brought some confusion, and contract tracers and local health authorities were left to find ways to persuade the community to participate in pandemic surveillance [36]. In addition, despite the initial goal of hiring local contact tracers to prioritize the most affected communities, the large number of cases overwhelmed the ChiTracing team, unable to match contact tracers with their own communities. Uncertainty and changing policies may have impeded the contact tracer’s ability to gain community trust, leading to the low rate of contact all completion. Notably, contact tracers transitioned to provide more targeted street outreach for the COVID-19 vaccine update in their respective communities [37]. Enhancing efforts to address mistrust in public health, and more broadly, government outreach efforts will improve community participation and adherence to public health interventions and policy guidelines [38].

## Conclusion

Despite the city-wide efforts, contact tracing in Chicago was slow and often incomplete. Telephone-driven contact tracing might not have been an effective way to deliver interventions, due to people’s hesitation to pick up the phone because of widespread telemarketing and fraud perpetrated via phone. To get around this issue, text and email messaging were employed in North Carolina, which was found to be more effective in timely outreach and isolation [35]. Given the ubiquity of COVID-19 in all aspects of people’s lives, it is also possible that some individuals may have taken steps on their own to get tested without participating in contact tracing.

We showed that individuals who were affected by the highest burden of the COVID-19 pandemic were less likely to engage in contact tracing. This pattern often holds for other public health interventions and initiatives. Efforts to build trusted messengers need to invest in community-based efforts to increase the trust of the public, which is a key to effective future contact tracing and other public health efforts.

Studies exploring barriers to participating in phone or computer-based public health interventions, such as mHealth or telehealth, can inform ways to improve intervention effectiveness for all demographic groups in a rapidly changing healthcare environment. Furthermore, strategies to increase the participation of conventionally underrepresented groups in clinical trials and public health interventions should continue to be explored for equitable healthcare.

## Supporting information

10.1017/cts.2026.10744.sm001 Kim et al. supplementary material Kim et al. supplementary material

## Supplementary material

The supplementary material for this article can be found at https://doi.org/10.1017/cts.2026.10744.
