# Supplementary material for: Community-engaged COVID-19 contact tracing initiative in Chicago
Source: J Clin Transl Sci. 2026 May 6;10(1):e88. doi: 10.1017/cts.2026.10744 (PMC13227127; doi:10.1017/cts.2026.10744)
Supplement: Kim et al. supplementary material [file S2059866126107444sup001.docx]

**Supplementary Table 1. Call disposition definition**

| **Call type** | **Label** | **Definition** |
| --- | --- | --- |
| **Completed** | Call completed | Interview, notice, or monitoring completed |
| **Refused** | Call Refused | Refused to be interviewed, notified, or monitored |
| **Incomplete** | Not reachable |  |
|  | Not reachable | Contact was not reachable |
|  | Busy/No answer | Received busy signal or did not pick up without a voicemail option |
|  | Incorrect number | Wrong or inactive phone number |
|  | No phone number | No phone number was provided |
|  | Potentially deceased | Contact potentially deceased |
|  | Hospitalized | Contact is hospitalized |
|  | Administrative closure | Call attempts more than 4 days or 3 calls with 5 hours between attempts |
| **Excluded** | Call pending |  |
|  | In progress | Case or contact interview is in progress |
|  | Call scheduled | A future call time has been scheduled |
|  | Callback scheduled | A call was placed back into a queue for action |
|  | Callback required | Required to callback |
|  | Callback/Call disconnected | Started the call with the contact but was disconnected or asked to call back |
|  | Left voicemail/message | Left a voicemail or a voice message |
|  | Out of Jurisdiction | Outside the Chicago jurisdiction |
|  | Duplicate record | Case or contact is duplicate |
|  | Referred to CDPH CST | Contact was identified as living in a congregate setting |

CDPH: Chicago Department of Public Health

CST: Congregate Settings Team
